# A Novel Method for Assessing Sinusitis Activity in Minimally Symptomatic Patients with ANCA-Associated Vasculitis: A Pilot Study

**DOI:** 10.3390/jcm14248972

**Published:** 2025-12-18

**Authors:** Michał S. Kaczmarczyk, Piotr Rot, Elżbieta Głuch, Maria Sobol, Arkadiusz Zegadło, Dariusz Jurkiewicz, Stanisław Niemczyk, Ksymena Leśniak

**Affiliations:** 1 Department of Otolaryngology and Laryngological Oncology with Clinical Department of Craniofacial Surgery, Military Institute of Medicine—National Research Institute, Szaserów 128, 04-349 Warsaw, Poland; 2 Department of Internal Diseases, Nephrology and Dialysis, Military Institute of Medicine—National Research Institute, Szaserów 128, 04-349 Warsaw, Poland; egluch@wim.mil.pl (E.G.); sniemczyk@wim.mil.pl (S.N.);; 3 Department of Biophysics, Physiology and Pathophysiology, Medical University of Warsaw, 02-901 Warsaw, Poland; maria.sobol@wum.edu.pl; 4 Clinical Department of Radiology, Military Institute of Medicine—National Research Institute, 04-141 Warsaw, Poland

**Keywords:** ANCA, sinusitis, computer tomography

## Abstract

**Objectives**: Antineutrophil cytoplasmic antibody (ANCA)-associated vasculitis (AAV) is a group of vasculitides sharing a common pathophysiology, which affects small and medium blood vessels. Sinonasal involvement is one of the most common manifestations of AAV. The goal of this study was to find the most suitable method to assess paranasal sinus changes in a group of patients with ANCA-associated vasculitis and renal involvement. Subjective scales like Lund–Mackay and Zinreich were compared with a three-dimensional (3D) volumetric method. Pre- and post-treatment computer tomography were compared. **Methods**: Computer tomography, nasal symptoms, and endoscopy of 28 patients hospitalized at the Department of Internal Diseases, Nephrology and Dialysis, Military Institute of Medicine—National Research Institute were assessed retrospectively. Paranasal sinus tomography was performed during treatment induction and after achieving disease remission (BVAS = 0) to assess treatment effectiveness. Radiological analysis was performed with the Lund–Mackay scoring system, Zinreich scoring system, and 3D volumetric scoring system with the usage of Slicer 3D analysis. The radiologic scoring systems were compared. **Results**: The statistically significant differences in treatment effectiveness were observed for the Zinreich scale on both the right and left side. Similar to the 3D volumetric scoring system, the right and left maxillary sinuses demonstrated statistically significant differences. On the other hand, no statistically significant differences were found between the first and second visits for the Lund–Mackay or total Global Osteitis scores on either side. The strongest correlation was achieved between the Zinreich scoring system and 3D volumetric scale. **Conclusions**: The three-dimensional CT volumetric analysis demonstrated higher SRM (standardized response mean) values than the Zinreich score on both sides, but the differences were not statistically significant. The Zinreich scoring system should be used instead of the Lund–Mackay scale in everyday clinical practice.

## 1. Introduction

Antineutrophil cytoplasmic antibody (ANCA)-associated vasculitis (AAV) includes granulomatosis with polyangiitis (GPA), microscopic polyangiitis (MPA), and eosinophilic granulomatosis with polyangiitis (EGPA). The prevalence of AAV varies worldwide, ranging from 187 to 210 cases per million inhabitants, showing an increase in America and Europe [1]. The introduction of immunosuppressive therapy and corticosteroids transformed GPA from a fatal disease into a chronic condition with remission achieved in up to 80% of patients. Nevertheless, despite significant therapeutic advances, relapse remains common, occurring in approximately 50% of patients within five years, thus necessitating long-term maintenance treatment and reliable markers of remission [2].

Sinonasal involvement is among the most frequent and earliest manifestations of AAV, especially in GPA. In the active phase, patients typically present with mucosal inflammation, granulations, crusting, or necrosis, while long-term disease progression may lead to septal perforation, turbinate loss, and saddle nose deformity [3]. Chronic sinonasal inflammation is also a major contributor to olfactory dysfunction, which often persists even after systemic disease control [4]. Despite the clinical importance of sinonasal changes, they remain nonspecific and may overlap with other inflammatory or infectious diseases, making radiologic evaluation an essential component of differential diagnosis and disease monitoring [5].

Computed tomography (CT) provides a comparatively noninvasive and reproducible means of evaluating sinonasal pathology. Scoring systems such as the Lund–Mackay staging system (LMSS), the Zinreich modification, and the Global Osteitis Scoring Scale (GOSS) are widely used to assess the extent of mucosal thickening, opacification, and bony changes. Moreover, modern three-dimensional (3D) volumetric analysis allows for the precise quantification of disease volume and progression, providing an objective correlate of disease severity [6,7,8,9].

There are multiple comparisons of paranasal sinus computer tomography scoring systems described in the literature. Chronic rhinosinusitis (CRS) is the most commonly assessed disease, but multisystem diseases with paranasal manifestation have been analyzed as well. The most frequently used scoring systems are Lund–Mackay and Zinreich (both the simplified and full version). Less common are the direct measurement of maximal mucosal thickness, the Nair scoring system, the Okushi scoring system, two-dimensional computerized analysis of a single coronal slice through the osteomeatal complex, and 3D volumetric paranasal sinus analysis. We found the following diseases with a comparison of scoring systems: cystic fibrosis, ciliary dyskinesia, odontogenic sinusitis, eosinophilic chronic rhinosinusitis, and systemic lupus erythematosus. In the latter, magnetic resonance imaging was used, as the authors point out its useability in controlling rheumatic diseases with paranasal sinus manifestations [10,11,12,13,14,15].

The present study aimed to retrospectively evaluate radiologic changes in paranasal sinuses among patients with AAV using multiple CT-based scoring systems to find the most appropriate scale for comparing sinusitis in orphan diseases and to compare radiological outcomes between patients treated with cyclophosphamide and rituximab (RTX) [5,9]. We hypothesized that the use of more precise and quantitative scoring systems was particularly advantageous in rare diseases like AAV.

## 2. Materials and Methods

### 2.1. Methodology

The inclusion criteria were at least two computed tomography scans of the paranasal sinuses and a diagnosis of ANCA small-vessel vasculitis. The first computed tomography scan was performed at the beginning of the treatment, and the second one was performed during the remission of vasculitis. Subsequently, each radiology imaging was assessed in the following manner: the Lund–Mackay staging system, the Zinreich staging system (a modification of Lund–Mackay), the Global Osteitis Scoring Scale, and the 3D volumetric scoring system method. Among the 42 patients enlisted in the study, 28 met the inclusion criteria. Ultimately, 56 computed tomography scans of the paranasal sinuses were assessed.

The Lund–Mackay staging system sets a value of 0, 1, or 2 to each of the frontal, maxillary, anterior ethmoid, posterior ethmoid, and sphenoid sinuses and a value of 0 or 2 for the OMC (the osteomeatal complex) depending on the occlusion. Score assignments are 0 if the sinus is clear, 1 if the sinus is partially opacified (in our case it scored 1 if about 50% of the sinus was opacified), and 2 if the sinus is completely opacified. The maximum score for each side is 12. The total maximum score is 24.

The Zinreich staging system, which is a modification of the Lund–Mackay staging system, increased the scale to a range from 0 to 5 for each sinus. Each sinus is assigned a score based on the percentage of opacification from mucosal thickening as follows: 0 = 0%, 1 = 1% to 25%, 2 = 26% to 50%, 3 = 51% to 75%, 4 = 76% to 99%, and 5 = 100% or completely occluded. The OMC is given a score of 0 to 2 depending on whether it is completely patent, partially obstructed, or completely obstructed. Then, the total outcome is summed up for each side separately, with the highest possible score being 54.

The Global Osteitis Scoring Scale [6] was used as the system for osteitis assessment. We decided to choose the GOSS because of its simplicity, similar to the Lund–Mackay staging system. Therefore, we used grading suggested by its developers.

The grading per sinus was as follows:

Grade 1: Less than 50% of the sinus walls involved and osteitis <3 mm wide.
Grade 2: Less than 50% of the sinus involved and 3–5 mm width of osteitic lesions.
Grade 3: Less than 50% of the sinus involved and wider than 5 mm or greater than 50% of the sinus wall involved and <3 mm wide osteitic lesions.
Grade 4: Greater than 50% of the sinus wall involved and 3–5 mm width of osteitic lesions.
Grade 5: Greater than 50% of the sinus wall and osteitic lesions thicker than 5 mm.

In this way, each sinus was given a score ranging from 0 to 5. The scores of all 10 sinuses (right and left frontal, anterior ethmoid, posterior ethmoid, maxillary, and sphenoid) were added, producing a global osteitis score (range: 0–50). Osteitis was classified as not significant (<5), mild (5–20), moderate (20–35), and severe (higher than 35).

Slicer software 5.6.1 (3D Slicer, https://www.slicer.org) [16] was used for 3D volumetric scoring. The 3D volumetric scoring system method used a method previously described in the literature and scientific papers [3,7]. Essentially, receiving a 3D model from a DICOM file is called segmentation, and it uses differences in Hounsfield units to distinguish tissues on tomography scans. Segmented 3D models are composed of voxels. Our workflow with the CT scans is described below. Firstly, each CT scan was transformed to be in line in every plane. This allows a reduction in mistakes and accelerating the process of segmentation. Our voxels were 0.046 mm^3^ cubes, and the Hounsfield units used for radiodensity measurements were −3000 to −300 for air volume and −300 to 300 for soft tissue volume. All slices were segmented with a semiautomated method using “Threshold”, “Surface cut”, “Islands”, and “Logical operators” modifiers from the “Segment Editor” tab. Once the entire sinus was segmented, the total volume, air volume, and the volume of disease could be calculated automatically with the use of the “Segment Statistic” modifier from the “Quantification” tab. We used the following equation to calculate the individual sinus total volume percentage of the disease: percentage of disease volume = (volume disease)/(volume disease + volume air) × 100%. The outcomes of segmentation were shown as percentages of opacification from mucosal thickening and the absolute data.

### 2.2. Statistical Analysis

All statistical calculations were performed using Statistica 13.0 software (Dell Software Inc., Round Rock, TX, USA) and Microsoft^®^ Excel version 16.89.1 (Microsoft Corporation, Redmond, WA, USA). Quantitative variables were summarized using descriptive statistics, including the mean, standard deviation, median, and range. The distribution of each variable was assessed for normality using the Shapiro–Wilk test.

As the variables were not normally distributed, the nonparametric Wilcoxon signed-rank test was used to evaluate the effect of treatment on the assessed variables. To assess the correlations between the Zinreich scale, the Lund–Mackay score, and the 3D volumetric measurements of the frontal, maxillary, anterior ethmoid, posterior ethmoid, and sphenoid sinuses on both the right and left sides, the Spearman’s rank correlation coefficient (rₛ) was calculated. The strength of correlation was interpreted as follows: very strong (0.90–1.00), strong (0.70–0.89), moderate (0.40–0.69), weak (0.10–0.39), and negligible (0.00–0.09).

Categorical data were expressed as frequencies and percentages, and comparisons between categorical independent variables were conducted using the chi-squared or Fisher’s exact test, as appropriate.

All statistical analyses were performed separately for the right and left sides. A *p*-value of less than 0.05 was considered statistically significant.

## 3. Results

### 3.1. Patients

This study included 28 patients hospitalized at the Department of Internal Diseases, Nephrology and Dialysis, Military Institute of Medicine—National Research Institute between December 2023 and September 2025. The mean age was 59 ± 25 years (median: 63 years; range: 21–82 years), and the cohort comprised 15 women (53.6%) and 13 men (46.4%). Microscopic polyangiitis (MPA) was diagnosed among 14 patients (51.9%), while granulomatosis with polyangiitis (GPA) was diagnosed among 13 (48.1%). Two types of regimens were used: the rituximab (RTX) protocol implemented in the RAVE trial (375 mg/m^2^ per week for 4 weeks), which was approved for the induction of remission in GPA and MPA in the European Union [17], or the two-dose protocol (1 g at weeks 0 and 2), approved for rheumatoid arthritis [18]. In severe kidney disease, a protocol combining rituximab infusions and two pulses of intravenous cyclophosphamide was used [19]. The analysis comprised both protocols as a single variable in the RTX treatment group. The pulse cyclophosphamide (CF) protocol used in the CYCLOPS study [20] was applied.

Twenty-five patients received RTX treatment (89.2%), while three were treated with CF (10.7%). Due to the low number of patients in the CF group, the effect of treatment type on the parameters analyzed could not be reliably assessed. Therefore, the study focused solely on comparing the results between the first and second visits following the treatment.

Patients were assessed with the BVAS/WG scale to monitor disease activity. Remission was defined by acquiring 0 points in the scoring system.

The mean Dose Length Product (DLP) per study was 206 ± 65 mGy·cm prior to treatment and 185 ± 52 mGy·cm following treatment. The mean interval between the first and second CT scans was 11.3 ± 5.4 months, with a median of 10 months (range 4.1–22.3 months). Magnetic resonance imaging (MRI) may be considered as an alternative to computed tomography (CT). Previous studies have demonstrated that MRI is superior to CT in differentiating pathological changes within the paranasal sinuses; however, it remains less accurate in delineating sinonasal anatomy. MRI examinations frequently yield a lower spatial resolution than CT due to a reduced number of acquired slices, largely attributable to the greater time and resource requirements needed for MRI to achieve a level of precision comparable to CT. Consequently, the use of MRI to assess osteomeatal complex patency or the extent of ethmoid cell involvement is generally impractical [15,21].

### 3.2. Treatment Effectiveness

**Figure 1:**
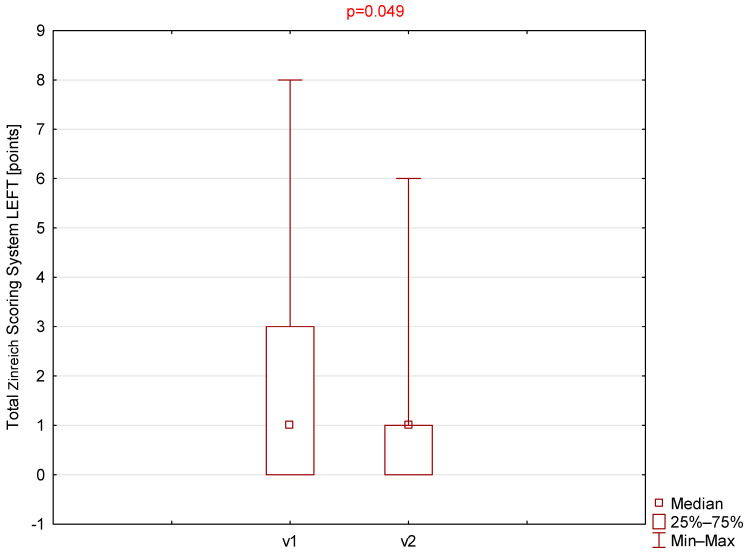
Comparison of total Zinreich scores (left side) between visit 1 (v1) and visit 2 (v2).

**Figure 2:**
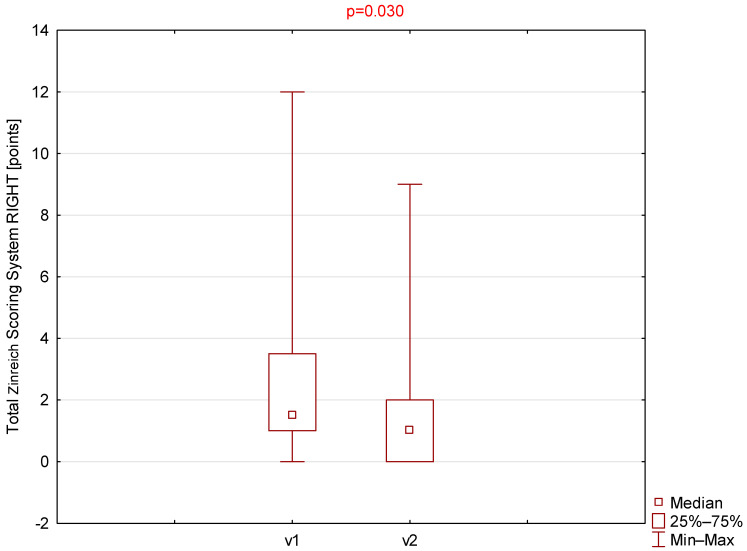
Comparison of total Zinreich scores (right side) between visit 1 (v1) and visit 2 (v2).

Statistically significant differences were observed for the Zinreich scale on both the right and left sides (Table 1, Figure 1 and Figure 2). The corresponding *p*-values were 0.030 and 0.049 for the right and left sides, respectively. No statistically significant differences were found between the first and second visits for the Lund–Mackay or total Global Osteitis scores on either side. The *p*-values were 0.151 and 0.180 for the right side and 0.173 and 1.000 for the left side for the LMSS and GOSS, respectively.

Although the prevalence of nasal symptoms, including epistaxis, headache, runny nose, nasal crusting or dryness, and nasal congestion, was lower during the second visit, the differences did not reach statistical significance (Table 2). Similarly, no statistically significant differences were demonstrated between the first and second visits for nasal obstruction, crusting, or rhinorrhea when analyzed separately for the right and left nasal cavities (Table 2).

**Figure 3:**
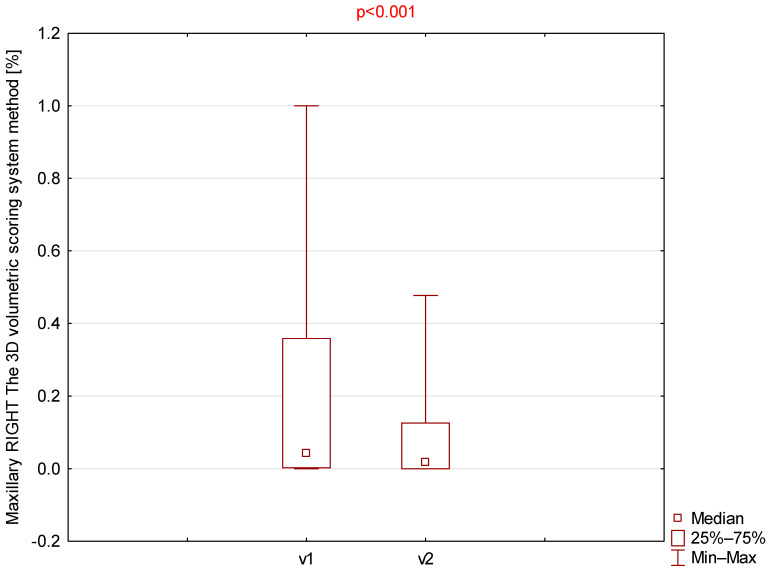
Comparison of right maxillary 3D volumetric scores between visit 1 (v1) and visit 2 (v2).

**Figure 4:**
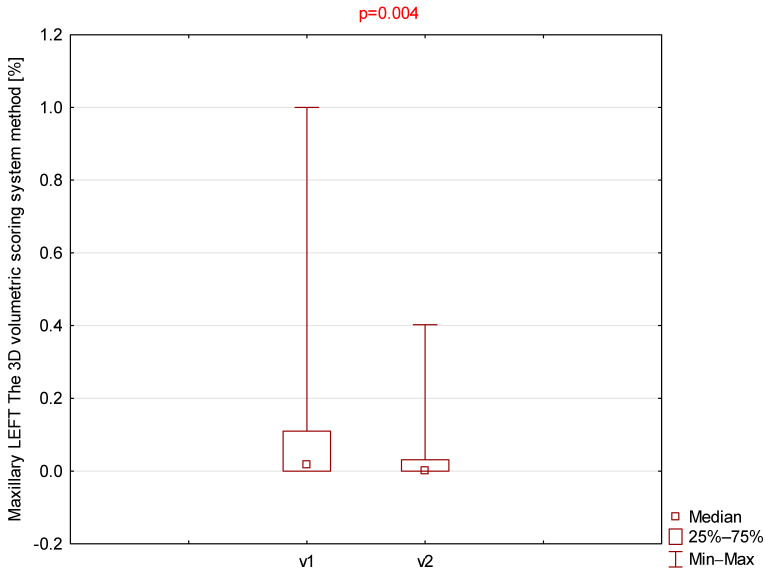
Comparison of left maxillary 3D volumetric scores between visit 1 (v1) and visit 2 (v2).

Among all parameters assessed using the 3D volumetric scoring system (Table 3), only the right and left maxillary sinuses demonstrated statistically significant differences (Figure 3 and Figure 4).

A statistically significant decrease in BVAS was observed between the two visits. The mean BVAS at the first visit was 6.9 ± 3.3 (median 6, range 1–14), whereas at the second visit it decreased to 0.5 ± 0.6 (median 0, range 0–2). A statistically significant reduction in BVAS was also noted when patients were stratified by ANCA-associated vasculitis subtype (GPA vs. MPA). In the GPA group, BVAS decreased from 6.9 ± 3.7 (median 6, range 1–14) at the first visit to 0.7 ± 0.7 (median 0, range 0–2) at the second visit. In the MPA group, BVAS decreased from 6.8 ± 3.0 (median 6, range 4–14) to 0.3 ± 0.5 (median 0, range 0–1).

**Figure 5:**
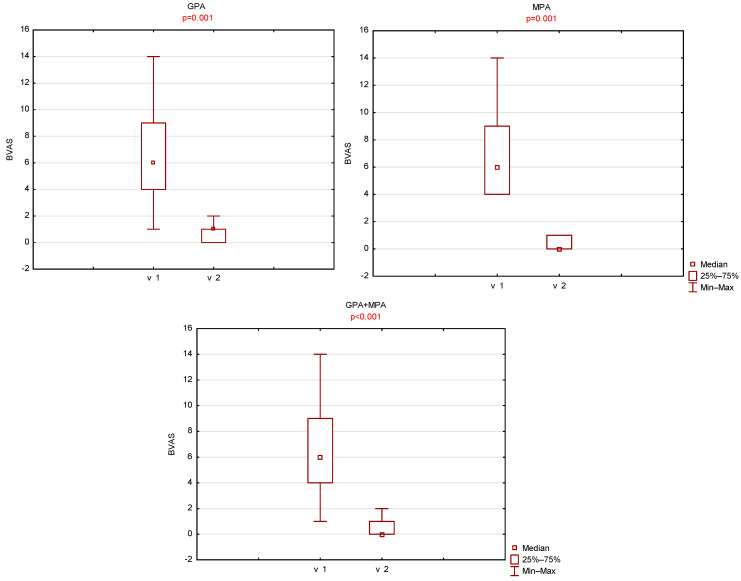
Comparison of left maxillary BVAS scores between visit 1 (v1) and visit 2 (v2).

No statistically significant differences in BVAS were found between the GPA and MPA groups at either visit. (Figure 5).

### 3.3. Correlation Between Scales

On the right side, statistically significant correlations were observed between the Zinreich and 3D volumetric scoring systems for all the analyzed variables (Table 4). The lowest correlation coefficient was revealed between the left posterior ethmoid Zinreich and left posterior ethmoid 3D variables (r = 0.438), indicating a moderate correlation. Stronger but still moderate correlations were at the right frontal, maxillary, anterior, and posterior ethmoid as well as the left maxillary sinus, whereas for the left anterior and both sphenoid sinuses, they were strong.

At the second visit, the correlation coefficients for both maxillary sinuses and both sphenoid variables remained strong to very strong. Although the correlation of both posterior ethmoids and the right anterior ethmoid increased, the coefficients for the left anterior sphenoid decreased to be borderline between moderate and strong (r = 0.694). We also obtained a strong association between the left frontal regions in the Zinreich and 3D volumetric methods (r = 0.72), whereas for the right frontal region there was no change.

On the right side regarding the Lund–Mackay scale and the 3D volumetric scoring systems, the correlations among the maxillary variables were statistically significant and strong at the first visit, whereas the posterior variable demonstrated a weak, non-significant correlation. In the remaining regions, the correlation coefficients indicated moderate associations. At the second visit (post-treatment), the correlation between the sphenoid region on the Lund–Mackay scale and the 3D volumetric measurements increased to r = 0.881, indicating a strong correlation, while the frontal region and the remaining variables showed moderate but statistically significant correlations (Table 4).

To assess whether three-dimensional CT volumetric analysis demonstrates greater sensitivity than traditional scoring systems in detecting subtle radiologic changes, we calculated the standardized response mean (SRM). The observed paired differences (3D−Zinreich) were 0.177 for the right side and 0.189 for the left side. The corresponding bootstrap mean differences were approximately 0.1681 and 0.171, and the 95% bootstrap confidence intervals were [−0.1782, 0.5264] and [−0.169, 0.493], respectively.

## 4. Discussion

This retrospective study evaluated radiologic changes in the paranasal sinuses of patients with AAV using multiple CT-based scoring systems—the Lund–Mackay staging system (LMSS), Zinreich modification, Global Osteitis Scoring Scale (GOSS), and three-dimensional (3D) volumetric analysis—and compared the outcomes between patients treated with cyclophosphamide and rituximab. The results revealed that while no statistically significant changes were observed in the classic CT-based scores, the volumetric 3D analysis revealed significant differences in the maxillary sinuses, suggesting that the 3D-based evaluation provided superior sensitivity for detecting subtle disease-related changes.

### 4.1. Radiologic Findings and Disease Activity

The absence of a significant improvement in the LMSS, Zinreich, and GOSS scores between the initial and follow-up CT scans suggests that sinonasal alterations in AAV often persist despite systemic remission. This finding aligns with previous reports emphasizing that chronic mucosal inflammation and osteitis might continue even after systemic disease control [5]. Studies by Tateyama et al. [3] and D’Anza et al. [5] showed that AAV-related sinonasal changes frequently progressed to structural damage, reflecting the destructive and fibrotic nature of the disease rather than active inflammation. In our study, differences between individual sinuses or even depending on the side were noticed. That differentiation may result from the anatomy of the specific paranasal sinuses or even nasal septum. It is well known that abnormalities like a deviated nasal septum, overgrown nasal concha, or anatomical varieties like Concha Bullosa and Haller cells can alter the nasal cavity ventilation and so have an impact on the withdrawal of inflammatory changes.

### 4.2. The Added Value of Three-Dimensional Volumetric Analysis

In contrast to traditional semi-quantitative scores, 3D volumetric analysis allowed for the detection of significant reductions in the volume of inflammatory tissue, particularly in the maxillary sinuses. This finding supports the notion that volumetric quantification offers enhanced precision and reproducibility in assessing sinonasal disease burden. Pallanch et al. [8] demonstrated that 3D CT analysis correlated more closely with objective measures of disease severity in chronic rhinosinusitis than the LMSS. Our results extended this concept to AAV, indicating that 3D volumetric CT could serve as a valuable imaging biomarker for monitoring disease progression and therapeutic response in this complex vasculitis. We propose that replacing the LMSS and its derivatives with 3D volumetric assessment is crucial, as the latter provides a more objective and quantifiable measure of disease burden. This impartiality of 3D segmentation allows the comparison of results worldwide, as the inconsistency of reporting radiologic data is still an issue now [5]. The main limitation of 3D volumetric analysis is its higher entry threshold compared to the conventional scoring systems mentioned above. In previous studies, it was outlined that 3D volumetric analysis required additional specialized software and was harder to master than the use of the LMSS or Zinreich system scales. Now, it is more accessible and easier to learn thanks to freeware applications like Slicer 3D and automatic or semiautomatic segmentation protocols. In our study, all segmentations were performed by an otolaryngologist resident under the supervision of an experienced radiologist. This study confirms its wider availability and encourages doctors of specialties other than radiology to use this tool. Moreover, thanks to artificial intelligence development, it is expected to make the process of 3D analysis fully automated [22,23,24]. Although the 3D volumetric measurements showed higher standardized response mean values than the Zinreich score on both sides, the confidence intervals included zero, indicating that these differences were not statistically significant at the 0.05 level. A larger study will be required to determine whether CT volumetric analysis is indeed more sensitive than traditional scoring systems such as the Zinreich score.

### 4.3. Comparison of Treatment Outcomes

No significant radiologic differences were identified between patients treated with cyclophosphamide and those receiving rituximab. However, as noted earlier, the cyclophosphamide group included only three patients, precluding a reliable statistical comparison between the treatment groups. Previous clinical trials and meta-analyses showed the comparable efficacy of both agents in inducing remission and preventing relapse in ANCA-associated vasculitis [25,26]. Terrier et al. [25] and the EULAR 2022 recommendations [26] confirmed that rituximab was at least as effective as cyclophosphamide, with a more favorable long-term safety profile. The lack of radiologic differentiation in our cohort likely reflected the irreversible character of sinonasal damage rather than therapeutic inefficacy.

### 4.4. Clinical–Radiologic Correlation

Despite radiologic assessments, no statistically significant changes were observed in sinonasal symptoms such as nasal obstruction, crusting, or epistaxis. Such a weak correlation between imaging findings and clinical manifestations has been well documented in GPA and chronic rhinosinusitis [5]. Persistent symptoms may result from scarring, mucosal atrophy, or deformity rather than ongoing inflammation. Hence, while CT imaging remains essential for monitoring structural disease, it should be interpreted alongside endoscopic and functional assessments, such as olfactory testing or patient-reported outcome measures.

### 4.5. Correlation Between Radiological Scales

It was found that both the Lund–Mackay and Zinreich scoring systems correlated with the 3D volumetric system. The overall correlations were stronger with the Zinreich scale, which tended to be more precise despite being subjective, which was consistent with studies conducted previously [7]. We believe that achieving a strong or very strong correlation between systems allows their alternate use, at least in clinical settings. Nevertheless, volumetric analysis should be used in every case of comparing data, especially by different researchers, for example in multicenter studies. Notably, we observed variability in the strength of correlations among individual sinuses and between sides. Although correlations between the left and right sides were generally moderate to strong, they were not uniform across all sinus regions. This asymmetry likely reflects anatomical and pathological heterogeneity within the paranasal sinuses, which is consistent with prior observations that inflammatory burden and ventilation patterns may differ by sinus and side. Such variability highlights the importance of evaluating each sinus compartment individually rather than relying solely on total or composite scores. It also suggests that certain sinus groups, particularly the sphenoid and anterior ethmoid cells, may exhibit more consistent structural and volumetric relationships, whereas maxillary and posterior ethmoid sinuses may be more susceptible to side-dependent variation.

### 4.6. Study Limitations

This study has several limitations that should be acknowledged. These include its retrospective, single-center design, a relatively small sample size, and heterogeneous follow-up duration. In particular, the cyclophosphamide-treated subgroup (*n* = 3) was too small to allow a reliable statistical comparison of treatment efficacy between therapeutic regimens. Variability in the intervals between CT scans may also have influenced volumetric measurements. Although all 3D volumetric segmentations were performed by an otolaryngology resident under the supervision of a radiologist, some degree of interobserver variability may not be excluded despite the use of validated scoring systems. Furthermore, while the 3D volumetric analysis demonstrated a greater sensitivity for detecting subtle disease-related changes, its practical application remains limited by the need for specialized software and technical expertise. Finally, the absence of quality-of-life or functional assessments, combined with reliance on retrospective symptom data, limits the clinical interpretability and correlation of radiologic findings.

## 5. Conclusions

Sinonasal alterations in AAV often persist despite systemic remission, reflecting chronic and partly irreversible damage. Three-dimensional CT volumetric analysis shows greater sensitivity than traditional scoring systems, particularly in the maxillary sinuses and anatomically complex regions. The Zinreich scale should be preferred over the Lund–Mackay system in routine practice, with 3D volumetric analysis serving as a valuable adjunct for the objective assessment of local disease activity. Although all three scoring methods correlate well, volumetric analysis provides the highest sensitivity and consistency. Future multicenter studies with standardized imaging protocols are needed to validate its role as an objective biomarker in AAV, establish volumetric thresholds, improve automation, and confirm its prognostic value.

## Figures and Tables

**Table 1:** Descriptive statistics (mean ± SD, median, range) for the Lund–Mackay, Zinreich, and total Global Osteitis scores at first (V1) and second (V2) visits, right and left sides.

	RIGHT								
	**v1**	**v2**							
	Total Lund–Mackay Scoring System	Total Lund-Mackay Scoring System	*p*	Total Zinreich Scoring System	Total Zinreich Scoring System	*p*	Total Global Osteitis Scoring Scale	Total Global Osteitis Scoring Scale	*p*
Mean ± SD	1.00 ± 1.72	0.61 ± 1.31		2.61 ± 3.07	1.61 ± 2.38		0.18 ± 0.94	0.29 ± 1.01	
Median (min–max)	0 (0–6)	0 (0–6)	0.151	1.5 (0–12)	1 (0–9)	0.030	0 (0–5)	0 (0–5)	0.180
	LEFT								
Mean ± SD	0.75 ± 1.53	0.43 ± 1.03		1.82 ± 2.29	0.93 ± 1.44		0.14 ± 0.59	0.14 ± 0.76	
Median (min–max)	0 (0–5)	0 (0–5)	0.173	1 (0–8)	1 (0–6)	0.049	0 (0–3)	0 (0–4)	1

**Table 2:** Comparison of the prevalence of nasal signs and symptoms between the first (V1) and second (V2) visits for the right and left sides.

**Visit**	**Epistaxis**			**Headache**			**Runny nose**		
	absent	present	*p*	absent	present	*p*	absent	present	*p*
v1	19	6	0.247	22	3	0.234	21	4	0.667
	76.0%	24.0%		88.0%	12.0%		84.0%	16.0%	
v2	22	2		24	0		22	2	
	91.7%	8.3%		100.0%	0.0%		91.7%	8.3%	
	**Dry nose**			**Nasal congestion**					

	absent	present		absent	present	1.0			
v1	19	6	0.098	21	4				
	76.0%	24.0%		84.0%	16.0%				
v2	23	1		21	3				
	95.8%	4.2%		87.5%	12.5%				
	**L Nasal obstruction**			**L Crusting**			**L Rhinorrhea**		
	absent	present	*p*	absent	present	*p*	absent	present	*p*
v1	19	6	0.716	20	5	0.213	16	9	0.300
	76.00%	24.00%		80.00%	20.00%		64.00%	36.00%	
v2	14	3		18	1		14	3	
	82.40%	17.70%		94.70%	5.30%		82.40%	17.70%	
	**R Nasal obstruction**			**R Crusting**			**R Rhinorrhea**		
v1	22	3	0.672	21	4	0.370	18	7	0.490
	88.00%	12.00%		84.00%	16.00%		72.00%	28.00%	
v2	14	3		18	1		14	3	
	82.40%	17.70%		94.70%	5.30%		82.40%	17.70%	

**Table 3:** Descriptive statistics (mean ± SD, median, and range) for the 3D volumetric scoring system scores at first (V1) and second (V2) visits, right and left sides.

	The 3D Volumetric Scoring System Method														
	Frontal			Maxillary			Anterior Ethmoid			Posterior Ethmoid			Sphenoid		
	v1	v2	*p*	v1	v2	*p*	v1	v2	*p*	v1	v2	*p*	v1	v2	*p*
RIGHT															
Mean ± SD [%]	3.9 ± 11.3	3.0 ± 11.8		21.0 ± 29	8.8 ± 14.1		6.1 ± 14.5	3.0 ± 8.5		6.8 ± 13.1	3.6 ± 13.6		3.1 ± 14.5	4.8 ± 16.4	
Median [%] (min–max)	0.0 (0–45.7)	0.0 (0–60.1)	0.715	4.2 (0–100)	1.9 (0–47.7)	<0.001	0.0 (0–60.3)	0.0 (0–35.9)	0.161	0.0 (0–44)	0.0 (0–61.2)	0.327	0.0 (0–77)	0.0 (0–70.7)	0.753
LEFT															
Mean ± SD [%]	0.6 ± 2.0	0.5 ± 1.9		12.1 ± 24.3	4.4 ± 9.4		4.3 ± 13.1	1.7 ± 4.6		4.4 ± 11.8	0.9 ± 4.7		1.7 ± 8.3	4.1 ± 13.1	
Median [%] (min–max)	0.0 (0–9.1)	0.0 (0–9.1)	0.285	1.8 (0–100)	0.2 (0–40.2)	0.004	0.0 (0–54.7)	0.0 (0–20)	0.249	0.0 (0–44)	0.0 (0–25)	0.091	0.0 (0–44)	0.0 (0–53)	0.249

**Table 4:** Spearman’s rank correlation coefficients between the Lund–Mackay and Zinreich scoring systems and 3D volumetric measurements before (v1) and after treatment (v2), left and right sides.

	Lund–Mackay and 3D Volumetric Scoring System Method				Zinreich and 3D Volumetric Scoring System Method			
	v1							
	L		R		L		R	
	r	*p*	r	*p*	r	*p*	r	*p*
Frontal	x	x	0.67	<0.001	x	x	0.531	0.005
Maxillary	0.542	0.003	0.750	<0.001	0.542	0.003	0.583	0.001
Anterior ethmoid	0.881	<0.001	0.648	<0.001	0.881	<0.001	0.489	0.008
Posterior ethmoid	0.438	0.020	0.375	0.054	0.438	0.020	0.480	0.01
Sphenoid	0.720	<0.0001	0.540	0.003	0.720	<0.0001	0.881	<0.001
	**V2**							
Frontal	x	x	0.531	0.005	0.720	<0.001	0.531	0.005
Maxillary	0.371	0.052	0.583	0.001	0.696	<0.001	0.918	<0.001
Anterior ethmoid	0.528	0.004	0.489	0.008	0.692	<0.001	0.759	<0.001
Posterior ethmoid	x	x	0.480	0.010	0.694	<0.001	0.817	<0.001
Sphenoid	0.733	<0.001	0.881	<0.001	0.998	<0.001	0.999	<0.001

## Data Availability

Data analyzed in study is present in the Department of Otolaryngology and the Department of Internal Diseases, Nephrology and Dialysis, Military Institute of Medicine, Warsaw.

## Abbreviations

The following abbreviations are used in this manuscript:

ANCA	antineutrophil cytoplasmic antibodies
AAV	ANCA-associated vasculitis
3D	three-dimensional
GPA	granulomatosis with polyangiitis
MPA	microscopic polyangiitis
EGPA	eosinophilic granulomatosis with polyangiitis
CT	computer tomography
LMSS	Lund–Mackay scoring system
GOSS	Global Osteitis Scoring Scale
CRS	chronic rhinosinusitis
MRI	magnetic resonance imaging
RTX	rituximab
CF	cyclophosphamide
OMC	the osteomeatal complex
DNS	deviated nasal septum
